# On spiking behaviour of oyster fungi Pleurotus djamor

**DOI:** 10.1038/s41598-018-26007-1

**Published:** 2018-05-18

**Authors:** Andrew Adamatzky

**Affiliations:** Unconventional Computing Lab, CSCT, UWE Bristol, UK

## Abstract

We recorded extra-cellular electrical potential of fruit bodies of oyster fungi Pleurotus djamor. We demonstrated that the fungi generate action potential like impulses of electrical potential. Trains of the spikes are observed. Two types of spiking activity are uncovered: high-frequency (period 2.6 min) and low-frequency (period 14 min); transitions between modes of spiking are illustrated. An electrical response of fruit bodies to short (5 sec) and long (60 sec) thermal stimulation with open flame is analysed in details. We show that non-stimulated fruit bodies of a cluster react to the thermal stimulation, with a single action-potential like spike, faster than the stimulated fruit body does.

## Introduction

Electricity is one of key factors shaping growth and development of fungi. Polarity and branching of mycelium are induced by electric fields^1^. Hyphae are polarised in electric fields^2^: sites of germ tube formation and branching, the direction of hyphal extension and the frequency of branching and germination could be affected by an electric field. Fungi also produce internal electrical currents and fields. Electrical current is generated by a hypha: positive current, more likely carried by protons^3^, enters tip of a growing hypha^4,5^. Current density reported is up to 0.6 *μ*A/cm^2^ ^3^. Electrostatic repulsion of charged basidiospores propulses the spores from alike charged basidium^6,7^. The electrical current can be involved or associated with translocation of material in pair with hydraulic pressure^8^. There are evidences of electrical current participation in the interactions between mycelium and plant roots during formation of mycorrhiza^9^.

In 1976 Slayman, Long and Gradmann discovered action potential like spikes using intra-cellular recording of mycelium of *Neurospora crassa*^10^. Four types of action potential have been identified: (1) spontaneous quasi-sinusoidal fluctuations of 10–20 mV amplitude, period 3–4 min, (2) as previous but shorter period of 20–30 sec, (3) cyanide induced oscillations of progressively lengthening period, starting with initial depolarisation of 20–60 mV, and (4) damped sinusoidal oscillations with amplitude 50–100 mV, period 0.2–2 mins. Twenty years later, Olsson and Hansson demonstrated spontaneous action potential like activity in a hypha of *Pleurotus ostreatus* and *Armillaria bulbosa*; they conducted intra-cellular recording with reference electrode in an agar substrate^11^. They shown that resting potential is −70 to −100 mV, amplitude of spikes varies from 5 to 50 mV, duration from 20 to 500 ms, frequency 0.5–5 Hz.

Olsson and Hansson shown that frequency of spiking increases in response to stimulating a hypha with a sulphuric acid, malt extract, water and fresh piece of wood. When stimulus is removed the frequency decreases and then increases again if the object is re-introduced. Olsson and Hansson^11^ speculated that electrical activity could be used for communication with message propagation speed 0.5 mm/sec. Changes in frequency of oscillations of a hypha in response to a wide range of stimuli reported in^11^ matches results of our personal studies with slime mould *Physarum polycephalum*, see overview in^12^. We established a mapping between volatile chemicals, wavelength of light and tactile stimulation, on one side, and changes in frequency of oscillations of electrical potential of slime mould’s protoplasmic tubes, on other side^13–16^; and, designed a prototype of a slime mould based sensor devices^17^. To advance our bio-sensing concepts to fungi and to evaluate a possibility of using wild fungi *in situ* as sensors we conducted experiments on electrical activity of fungi in conditions more close to natural conditions than experiments^10,11^ on intra-cellular recording of a hypha conducted in laboratory conditions with mycelium growing on a nutrient agar substrate. For our experiments, we chosen oyster mushrooms, species *Pleurotus*, family *Tricholomataceae*, they are most widely cultivated family of fungi^18^ with proven medicinal properties^19^, and they are amongst few species of carnivorous mushrooms^20^ which might add some unusual sensing properties. The paper is structured as follows. We describe experimental setup in Sect. 2. We characterise spontaneous spiking behaviour of fruit bodies in Sect. 3.1 and the fruits’ response to stimulation in Sect. 3.2. We reflect on results of the experiments in Sect. 4.

## Methods

We used commercial mushroom growing kits (© Espresso Mushroom Company, Brighton, UK) of pink oyster mushrooms *Pleurotus djamor*. Each substrate’s bag was 22 cm by 10 cm by 10 cm, 800–900 g in weight. The bag was cross-sliced 10 cm vertical and 8 cm horizontal and placed in a cardboard box with 8 cm by 10 cm opening. The fungi kits were kept at room temperature in constant (24 hr) ambient lighting of 10 lux.

**Figure 1:**
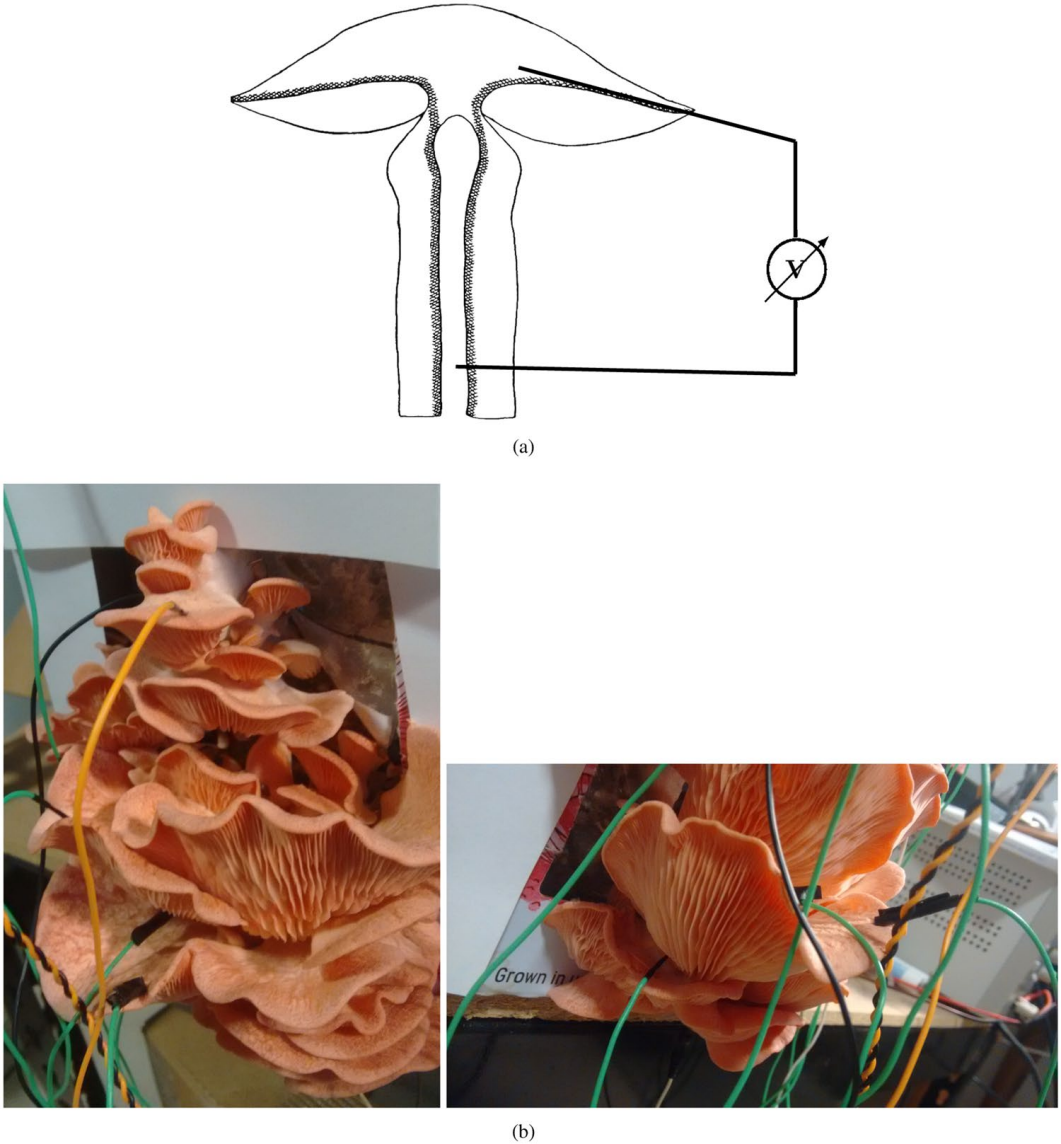
Experimental setup. (**a**) Position of electrodes in relation to translocation zone, cross-section of a fruit body showing translocation zone, drawing by Schütte^30^. (**b**) Photographs of fruit bodies with electrodes inserted.

Electrical potential of fruit bodies was recorded from the second-third day of their emergence. Resistance between cap and stalk of a fruit body was 1.5 MΩ in average, between any two fruits in the cluster 2 MΩ (measured by Fluke 8846A). We recorded electrical potential difference between cap and stalk of the fruit body. We used subdermal needle electrodes with twisted cable (© SPES MEDICA SRL Via Buccari 21 16153 Genova, Italy). Recording electrode was inserted into stalk and reference electrode in the translocation zone of the cap (Fig. 1a); distance between electrodes was 3–5 cm. In each cluster we recorded 4–6 fruit bodies simultaneously (Fig. 1b,c) for 2–3 days.

Electrical activity of fruit bodies was recorded with ADC-24 High Resolution Data Logger (Pico Technology, St Neots, Cambridgeshire, UK). The data logger ADC-24 employs differential inputs, galvanic isolation and software-selectable sample rates all contribute to a superior noise-free resolution; its 24-bit A/D converted maintains a gain error of 0.1%. Its input impedance is 2 MΩ for differential inputs, and offset error is 36 *μ*V in ±1250 mV range use. We recorded electrical activity one sample per second; during the recording the logger makes as many measurements as possible (typically up 600) per second then saves average value.

## Results

Here we provide evidence that fruit bodies exhibit spontaneous spiking behaviour, we also characterise types of trains of spikes observed. When calling the spikes spontaneous we mean they are not invoked by an intentional external stimulation, i.e. not expected by an external observer. Otherwise, the spikes indeed reflect physiological and morphological processes ongoing in mycelial networks and growing fruit bodies. We also provide evidence that fruit bodies respond to external stimulation by changing its electrical potential and that neighbours of stimulated fruit bodies might show action-potential like response.

### Spontaneous spiking

**Figure 2:**
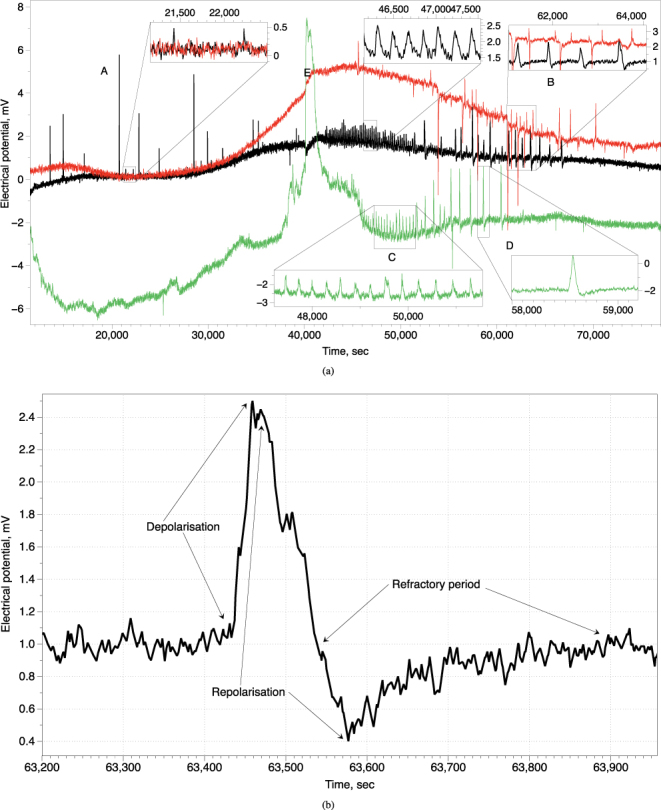
Electrical activity of fruit bodies. (**a**) Example of a dynamics of electrical potentials recorded from three fruit bodies of the same cluster during 70 K sec, c. 19 hours. Some modes of spiking activity are zoomed in the inserts. (**b**) Analysis of a spike in terms of action potential.

The electrical activity of fruit bodies shows a rich combination of slow (hours) drift of base electrical potential combined with relatively fasts (minutes) oscillations of the potential, see example at Fig. 2a. We observed trains of spikes of electrical potential. Each spike resembles an action potential where all ‘classical’ parts can be found (Fig. 2b): depolarisation, repolarisation and refractory period. The exemplar spike shown in (Fig. 2b) has a period of 130 sec, from base level potential to refractory-like period, depolarisation rate 0.05 mV/sec, repolarisation rate is 0.02 mV/sec, refractory period is c. 360 sec.

**Figure 3:**
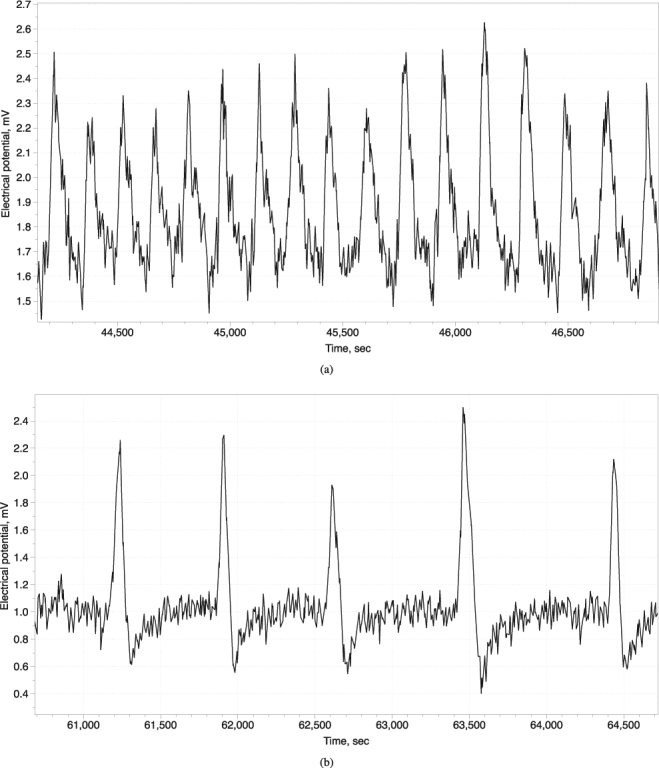
Examples of spike trains. (**a**) High frequency. (**b**) Low frequency.

**Figure 4:**
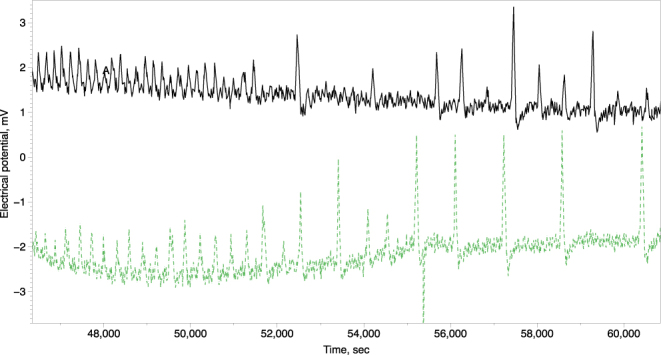
Example of transition from a train of H-spikes to a train of L-spikes. Electrical potential of two fruit bodies of the same cluster are shown by black solid and green dashed lines.

We observed two types of spike trains: high-frequency (H-spikes), a spike per c. 2.6 min, and low-frequency (L-spikes), a spike per c. 14 min, see examples in Fig. 3. Characteristics of the spikes are shown in Table 1. Period of L-spike is five time longer than period of H-spike. Amplitudes of H-spikes are just below 1 mV and of L-spikes is nearly 1.5 mV. Durations and depolarisation rates of L- and H-spikes are nearly the same. A repolarisation rate of L-spikes is a double of the repolarisation rate of H-spikes. Refractory period of L-spikes is ten times longer than the period of H-spikes. Trains of H-spikes last for up to two-and-half hours while trains of L-spikes for up to six hours. Transitions between trains of H-spikes and L-spikes have been also observed, see example in Fig. 4. Variability of the spikes’ periods, as expressed via standard deviations, are c. 10% of period for H-spikes and c. 20% for L-spikes; the amplitude 15% for H-spikes and L-spikes. Variability of depolarisation rates are c. 30% for H-spikes and c. 40% for L-spikes.

**Table 1:** Average characteristics of spikes.

Parameters	High frequency spikes	Low frequency spikes
Period, sec	160.5 (15.1)	838.8 (147.2)
Amplitude, mV	0.88 (0.14)	1.3 (0.21)
Duration, sec	115.5 (28.1)	142.6 (33.1)
Depolarisaton rate, mV/sec	0.022 (0.006)	0.025 (0.01)
Repolarisation rate, mV/sec	0.012 (0.002)	0.024 (0.007)
Refractory like period, sec	25.5 (4.2)	256.2 (80.6)
Duration of spike trains, min	80–150	130–360

Average values are indicated.

### Response to stimulation

**Figure 5:**
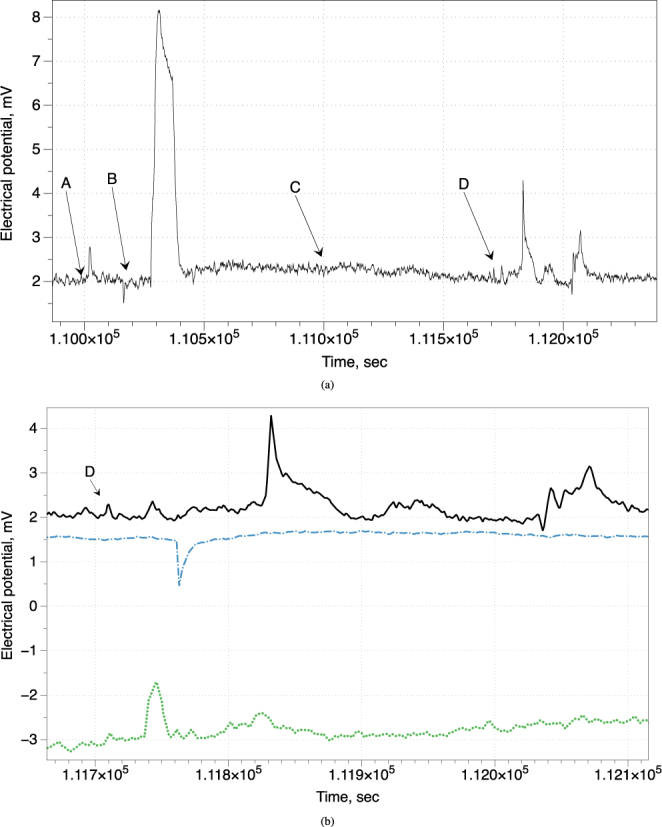
(**a**) Response to spirit (moment of application is shown by arrow labelled A), water (B), polydimethylsiloxane (C), thermal stimulation with open flame for 5 seconds (D). Time between two vertical lines is 500 sec. (**b**) Response to thermal stimulation: edge was burned for c. 5 sec. Potential of the stimulated fungi is shown by solid black line, two other members of the cluster by green dotted and blue dashed lines. Time between two vertical lines is 100 sec.

To check if there will be any changes in electrical potential in response to stimulation we applied 50 *μ*L of 40% ethanol, tap water, polydimethylsiloxane on top of fruit bodies’ caps and thermally stimulated edges of the caps with open flame for 5 sec. Exemplar responses are shown in Fig. 5. Stimuli were applied in 3 hr intervals. All stimuli but polydimethylsiloxane cause positive spike-like responses, parameters are shown in Table 2. There was no response to a drop of polydimethylsiloxane. A fruit body responded to application of water with nearly immediate negative spike with amplitude 0.43 mV and duration 8.2 sec, followed (after c. 98 sec) by a large positive spike, more likely due to change in capacitance.

**Table 2:** Parameters of a fruit body response to stimulation.

Stimulus	Amplitude, mV	Depolarisaton rate, mV/sec	Repolarisation rate, mV/sec	Duration, sec
water	6.1	0.2	0.05	141.5
spirit	0.8	0.03	0.02	51.2
thermal	2.1	0.1	0.03	99

Response of non-stimulated fruit bodies to the short-term thermal stimulation of a member of their cluster is shown in Fig. 5b. While the stimulated fruit body responded to a thermal stimulation after c. 103 sec delay, two other fruit bodies in the same cluster shown shorter latency times of their responses. One body responded with a positive spike 26 sec after stimulation (green dotted line in Fig. 5b), amplitude c. 1.2 mV, duration 21 sec. Another body responded with a negative spike 51 sec after stimulation (blue dash-dot line in Fig. 5b), amplitude c. 1 mV, duration 26.3 sec.

**Figure 6:**
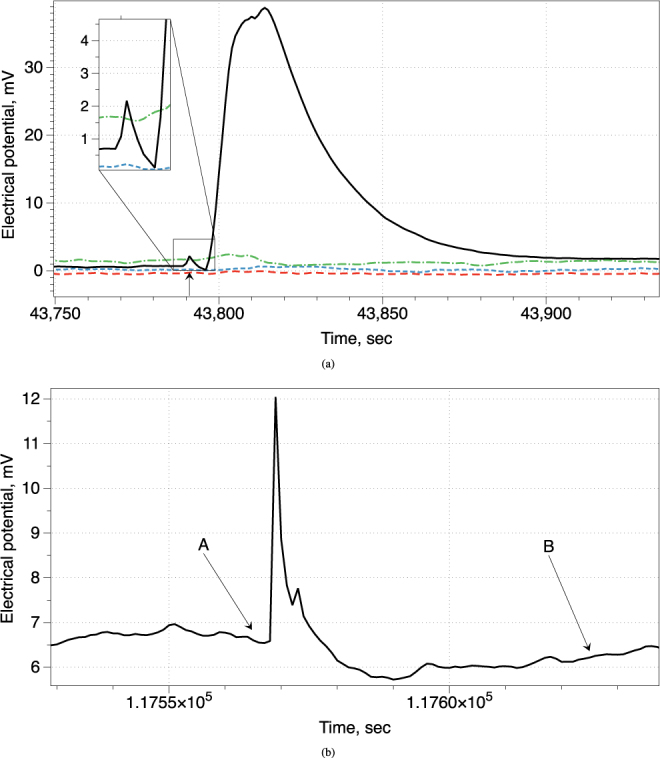
Response to a long-term thermal stimulation. Electrical potential measured on stimulated fruit body is shown by solid black line. Time between two ticks on horizontal axes on both plots is 50 sec. (**a**) Moment when stimulation was stopped is shown by arrow. Initial action-potential like response is magnified in the insert. (**b**) Response of an intact fruit body to stimulation of its distant neighbour, c. 6 cm away, by an open flame for c. 60 sec. Start of stimulation is show by arrow ‘A’ and end of stimulation by arrow ‘B’.

The fruit bodies’ response to a long term thermal stimulation–subjecting an edge of a cap to an open flame for 60 sec, was demonstrated to be highly pronounced. A typical response is shown in Fig. 6a. At first we observe an action potential like response of the stimulated fruit body, with depolarisation up c. 1.4 mV, followed by repolarisation by 2 mV. This response lasts 7.6 sec. It is immediately followed by a high-amplitude depolarisation. There electrical potential grows by 38.2 mV in 18.4 sec followed by slow repolarisation and returning to the base potential in 83 sec. Other fruit bodies react with short-living spikes to the long-term thermal stimulation of a member of their cluster. Example is shown in Fig. 6b. Four seconds after start of the stimulation, there is a sharp depolarisation by 5.2 mV reached in 1.38 sec. It follows by repolarisation by 6.2 mV reached in 20 sec. There is an indication of a refractory period c. 59 sec. The potential returns closely to its base (for this fruit body) level after stimulation ends.

## Discussion

We demonstrated that fruit bodies of oyster fungi *Pleurotus djamor* exhibit trains of action-like spikes of extracellularly recorded electrical potential. We observed two types of spikes: high-frequency spikes, duration nearly 3 min, and low-frequency spikes, duration nearly 14 min. The spikes are observed in trains of 10–30 spikes. The depolarisation and repolarisation rates of both types of spikes are the same. Refractory period of a high-frequency spike is one sixth of the spike’s period, and of a low-frequency spike one third of the spike’s period. We shown that fruit bodies respond with spikes of electrical potential in response to physical, chemical and thermal stimulation; not only a stimulated body responds with a spike but other fruit bodies of the cluster respond as well. These results might lay a foundation for studies of sensing and collective information processing in *Agaricomycetes*.

Fruit bodes, stromata, are made of interwoven hyphae, organic continuation of a mycelium. Thus by inserting electrodes in cap and stalk we measured extracellular electrical potential difference between the cap and the stalk as generated by interwoven hyphae. We observed trains of action potential like spikes. Microtubule bundles observed in basidiomycetae^21^ may be responsible for propagation of trains of action potential like spikes. Amplitudes of spikes measured were very low comparing to amplitudes reported in^10,11^ because the works cited used intra-cellular recording while we used extra-cellular.

Periods of spikes evidenced in^10^ 0.2–2 min, are comparable with period 2.5–3 min of high frequency spikes in our experiments. High frequency of oscillations of oyster fungi also similar to that recorded in slime mould of *Physarum polycephalum*: electrical potential between two electrodes connected by a protoplasmic tube oscillates with period 1–2 min^22–25^. In slime mould the calcium waves are reflected in oscillations of external membrane potential and periodic reversing of cytoplasmic flow in the tubes. Drawing up analogies between the slime mould and mycelial fungi we speculate that trains of spikes recorded in fruit bodies correlate, or even responsible for, translocation of nutrients, relocation of products of metabolism and communication. There are indications, see e.g. timing of spikes recorded from two fruit bodies of the same cluster in Fig. 4, that trains of spikes are coming from the mycelium, where they most likely originate in a way similar to calcium waves in slime mould *P. polycephalum*.

With regards to communicative function of the spikes, fungi responds to stimulation with singular spikes of electrical potential in their fruit bodies. Amplitude of the response is higher in the stimulated body than in its non-stimulated neighbours. However, non-stimulated members of the cluster respond earlier to the stimulation than the stimulated body itself. These response of the non-stimulated bodies to a destructive stimulation of one of the cluster’s members might be seen either as a ‘byproduct’ of an electrical potential deviations propagating from the damaged body through the mycelium network towards intact bodies or a purposeful signal to the intact bodies aimed at speeding up their growth and maturation to shorten a period leading to accelerated production of spores. As shown in^26^ (cited by^27^) sporulation could be induced by partial dessication, and a number of fruit bodies could be larger in proximity of injury. This observation is in line with finding that damaged mycelium responds with branching^28^; and, similar to sprouting response of a slime mould *P. polycephalum* to a dissection of its protoplasmic tubes^29^.
